# Protists—the dark matter of eukaryotic evolution

**DOI:** 10.1007/s00709-025-02066-w

**Published:** 2025-04-09

**Authors:** Peter Nick

**Affiliations:** Joseph Gottlieb Kölreuter Institute for Plant Sciences, Karlsruhe Institute of Technology, Karlsruhe, Germany

The vast majority of our universe consists of invisible matter. Since most analysis is based on detecting radiation, this so-called dark matter has remained inaccessible to investigation (Arbey 2021). This does not mean at all that dark matter is not relevant. In terms of total mass, dark matter represents 80% of the universe and has, therefore, tremendous impact. Dark matter is also familiar to biology. The majority of prokaryotes is still recalcitrant to cultivation, which makes it hard to study them. The term microbial dark matter has been coined for these invisible part of microbiology (for a critical appraisal of this term see Bernard et al. 2018). Meanwhile, methodological progress in sequencing made it possible to reconstruct the genomes of such microbes from DNA directly extracted from environmental samples, even though no one has ever seen these organisms in culture. This metagenomics has allowed to shed light into the obscurity of prokaryotic dark matter. However, the reconstruction of eukaryotic genomes is far more challenging, and we have to be aware that there may be a substantial amount of eukaryotic dark matter out there as well that has remained opaque so far. The mysterious world of eukaryotic protists still hides numerous secrets, and we need direct observation to unveil them, because some of these protists are far away from any of our model organisms, either, because they are surviving witnesses from ancient stages of evolution, or, because they have parted from the rest moving along very particularly evolutionary paths over a long time. To disentangle, the dark matter of eukaryotic evolution represents a complex task. Here, genomics alone will not do, genetic data need to be complemented by cellular details, knowledge on the ecology and physiology, and the ability to distil working hypotheses from a complex and often still incomplete description of traits. A milestone of this art was the legacy of the late Cavallier-Smith, published in this journal a few years ago (Cavalier-Smith 2021). Three contributions to the current issue take up the challenge and report novel and unexpected facets of eukaryotic protists.

Amoeba were long considered basal form of eukaryotic life, but meanwhile it has become clear that quite different life forms can pass through amoeboid stages during their ontogeny. Likewise amoeboid forms have evolved from different ancestral states. The Archamoebae are among the most mysterious amoebas. The genus *Pelomyxa* forms giant cells with remnants of flagellae that have lost motility and numerous nuclei. These cells also harbour mysterious organelles that are not surrounded by a membrane, and might result from RNA–protein condensate. The contribution to the current issue by Bogolyubov et al. (2025) investigates these organelles that accumulate nucleolin, a protein that is typical for the nucleolus all over the eukaryotes, and is involved in numerous RNA-related processes from rDNA transcription, maturation, and transport into the cytoplasm. These enigmatic organelles are not the only cellular peculiarity of *Pelomyxa*. Living in anoxic environments, they lack mitochondria, but instead keep different prokaryotic endosymbionts and form numerous glycogen granules thought to result from specific changes of anaerobic carbohydrate catabolism (Chistyakova et al. 2020). Electron microscopy suggests that the nucleolin-rich cytoplasmic organelles are coated by these granules. Overall, the anoxic lifestyle of these exotic eukaryotes has not only shaped their metabolism, but led to a cellular structure that is, to put it mildly, completely unorthodox.

Also, the contribution by Safonov et al. (2025) deals with amoeba; however, this time from a completely different group of organisms, the Dictyochophycea, belonging to a group of algae called Heterokontophyta comprising organisms as different as kelps, but also the Oomycetes (formerly mistaken as fungi). The unicellular *Rhizochromulina marina*, single species of its genus, can switch between an ameboid and a flagellate lifestyle. Similar to real amoeba, they can form long pseudopodia, which can fuse different cells into common networks, called meroplasmodia. The similarity with the Amoebozoa is superficial. While their pseudopodia are driven by actin (Grębecki 1990; Pomorski et al. 2007), actin seems to be peripheral in *R. marina*. Instead, the pseudopodia contain mainly microtubules. This curious organism has been discovered in the Mediterranean Sea, but the authors found it in the Arctic Sea. To get insight into the mobility of R. marina, the authors eliminated either microtubules with Nocodazole, or actin filaments with Latrunculin B, or used a combination of both drugs and tracked then the movement by video, using the trajectories for mathematical modelling. While the movement was impaired by either of the two drugs, the combination, unexpectedly, led to a promotion, indicative of cytoskeletal pools differing in dynamics playing antagonistic roles in motility. On a more general level, this case demonstrates again that it is dangerous to infer homology from superficial similarity, especially if dealing with phenomenon that are separated by a large evolutionary distance. There is no alternative to remain open-minded, establishing the details from the scratch, which is, what the authors did.

One driver for the genesis of eukaryotic cell has been the endosymbiotic uptake and domestication, a process that is partially continuing to proceed. A famous example is the associating between ciliates of the *Paramecium bursaria* complex and unicellular algae from the genus *Chlorella*. The contribution by Sabaneyeva et al. (2025) uncovers novel and unexpected facets of this paradigmatic case of endosymbiosis. They detect that a natural population for one species of this ciliate complex, *P. tritobursaria* keep the yeast *Rhodotorula mucilaginosa* as endosymbiont. They can replicate this association in the laboratory by feeding this yeast to endosymbiont-free host cells and suggest that the yeasts are kept as back-up for times of starvation. Since this yeast can act as human pathogen in patients with compromised immune system, this curious case of endosymbiosis is also of medicinal importance. The cellular details of yeast endosymbiosis match those of the usual zoochlorellae, for instance with respect to the presence of a perisymbiotic vacuole. This specific structure represents a secondary stage—first, the endosymbiont is swallowed as prey including the formation of a digestive vacuole from acidosomes and lysosomes (Kodama and Fujishima 2005). In a second step, this digestive vacuole buds off a smaller vacuole with the endosymbiont, accompanied by changes in membrane composition. This perialgal membrane wards off the fusion of further digestive vesicles (Kodama and Sumita 2021). Among the Chlorophytes, the conventional endosymbiont, *Chlorella*, is unusual by the presence of chitin in its cell wall (Kapaun and Reisser 1995), possibly in consequence of viral infection (Kawasaki et al. 2002). Thus, the unusual uptake of a yeast instead of *Chlorella* might be linked with the shared molecular composition of their cell walls.

These three glimpses into the dark matter of eukaryotic protists reveal unexpected and partially bizarre details from the early evolution of eukaryotes. These details seem so singular that it is difficult to link them to that what we can read in the textbooks. However, we have to keep in mind that most of the knowledge described in our textbooks derives from a fairly limited number of model organisms. They are model organisms in the first place, because they are experimentally accessible at ease. We claim that they are also models in the sense that they represent an entire group of organisms. While model organisms have been a very powerful tool for biology and helped to unveil universalities of life, we should never forget that these model organisms are something like a small area lit by the beam of our flashlight called science. To conclude that this still limited well-lit area tells us what is lingering in the dark would be naïve and misleading. The novel tools deriving from the work with model organisms, for instance, the genomic information, can now help to move forward into the dark. We have to do this keeping an open mind, because that what we will find there, can very well be very different and unfamiliar.
